# Salt Effect on Donnan Equilibrium in Montmorillonite Demonstrated with Molecular Dynamics Simulations

**DOI:** 10.1021/acs.jpcb.2c04016

**Published:** 2022-10-24

**Authors:** Ya-Wen Hsiao, Magnus Hedström

**Affiliations:** † Scientific Computing Department, STFC Daresbury Laboratory, Daresbury WA4 4AD, U.K.; ‡ Clay Technology, Ideon Science Park, SE-223 70 Lund, Sweden

## Abstract

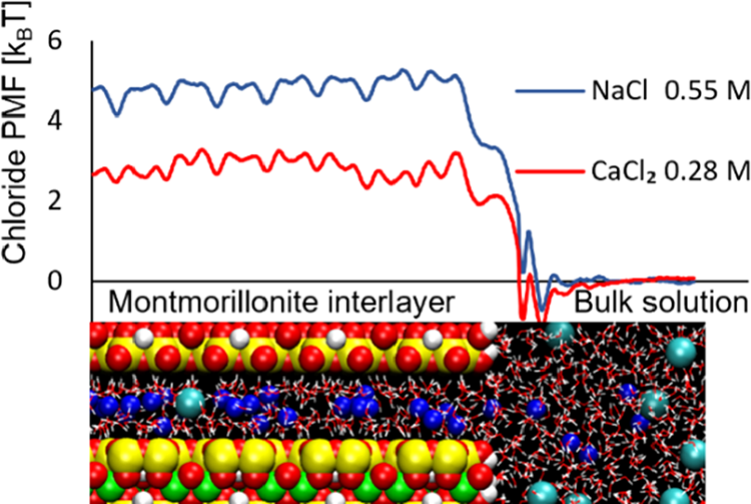

Donnan equilibrium governs the distribution of ions in many systems such as ion exchange membranes and biological cells in contact with an external electrolyte. Herein, Donnan equilibrium between bulk salt solution and bihydrated montmorillonite was investigated because such a system is of great importance for many nuclear waste disposal concepts. Specifically, we used molecular dynamics simulations to determine the partition coefficient of chloride, which was achieved by calculating the free-energy difference of chloride in the interlayer and the bulk using enhanced sampling methodology. Montmorillonite in equilibrium with either NaCl or CaCl_2_ was examined to elucidate the general difference between 1:1 and 2:1 salts. The concentration dependence of the partition coefficient for each salt was determined using three and four concentrations for NaCl and CaCl_2_, respectively. In the case of NaCl, we found that the partition coefficient increased linearly with the concentration, while for CaCl_2_, the increase was proportional to the square root of the concentration. A derivation of the partition coefficient using general Donnan theory that includes excess free energy contributions beyond the electrostatic Donnan potential is also presented. For both salts, the agreement between the partition coefficient from the simulations and Donnan theory was excellent. Although Donnan theory is a continuum theory derived without any reference to atomistic details, the present results justify its application to systems with nanoscale pores.

## Introduction

Bentonite clay is proposed as a barrier material in several geological disposal facility (GDF) concepts of high-level radioactive waste, and an accurate description of this material is of utmost importance given that the operating time for such GDFs may be up to one million years.^1^ For disposal concepts relying on canister containment,^2−4^ the bentonite buffer surrounding the canisters should develop a sufficiently high osmotic pressure to suppress sulfate-reducing bacteria.^5^ The bentonite buffer should also have a low hydraulic conductivity to ensure that any transport of corrosive agents, notably HS^–^, toward the canister is dominated by diffusion, which is a slow process. The desirable properties of bentonite originate from smectite clay minerals, in particular montmorillonite (Mt). Mt consists of aluminosilicate layers with lateral dimensions in the range of 50–500 nm and thickness ∼ 1 nm.^6,7^ Each layer has two tetrahedral silicon oxide sheets sandwiching an octahedral aluminum oxide sheet. The layers have a permanent negative charge due to isomorphous substitutions in the crystal structure and for Mt predominantly in the octahedral sheet.^8^ This permanent charge is compensated by exchangeable cations in the interlayer space, between adjacent particles. The specific configuration of layer charge and cations gives the interlayer a large affinity for water, which, in turn, is the origin of swelling when the clay has access to an external water source.^9−13^ The chemical potential of the external water controls the extent of swelling, and water can be added to/removed from the interlayer by changing the chemical potential. Thus, for Mt and other smectites, the solid volume fraction can vary, making them different from, for example, zeolites having a fixed micro-porosity.^14^ For unconfined Mt at a low water content, the change in layer separation is stepwise, one water layer (WL) at a time, seen by X-ray diffraction as discrete jumps of about 3 Å in the basal reflection, *d*_001_.^15−19^ On the other hand, Mt confined in a fixed volume develops an osmotic pressure, also called swelling pressure,^11,12,20^ when in contact with an external aqueous solution to ensure equal water chemical potential in both phases.^10−12,21^ Limiting the volume of the Mt-rich phase requires a semipermeable component, which withstands the swelling pressure and prevents transport of clay layers into the external bulk solution but allows the passage of water and ions between the two coexisting phases. In laboratory tests, a filter stone or a metal filter with sufficiently small pore size can serve as a semipermeable component. In a GDF, the host rock itself confines the bentonite, and the external bulk solution is present in fractures in the rock.

The equilibrium situation described above is called Donnan equilibrium^22,23^ and is, in addition to swelling clays, applicable to, for example, ion-exchange resins and membranes^24^ and to cell membranes.^25^ Apart from the formation of an osmotic pressure, systems in Donnan equilibrium also show an uneven distribution of ions between the two phases and an electric potential difference. Changes in the salinity of the external bulk solution will alter the Donnan equilibrium, affecting the osmotic pressure, ion distribution, and the electric potential difference.

**Figure 1:**
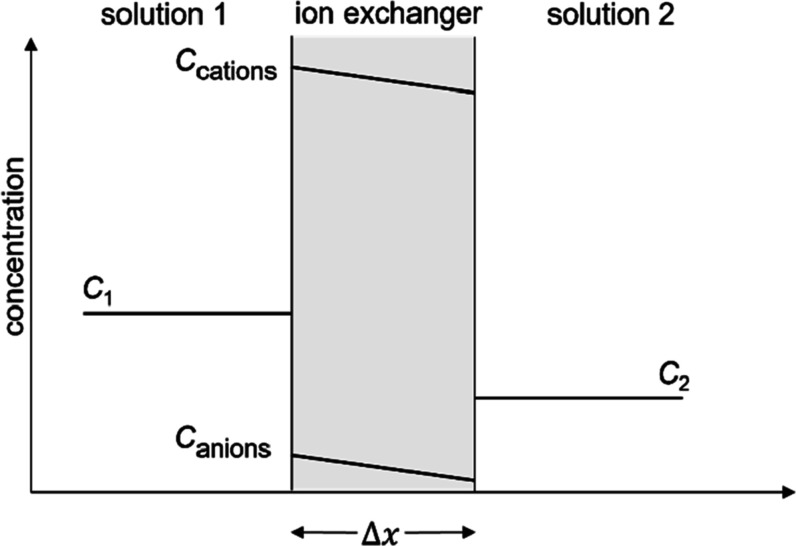
Schematic view of salt diffusion through a cation-exchange membrane in the ss. Because of Donnan equilibrium, there are jumps in the concentration at the interfaces between the bulk solutions and ion exchanger. On the macroscopic scale (length ≫ Debye length), the concentration appears to be discontinuous at the interfaces.

The uneven distribution of ions can be quantified by the partition coefficient Ξ, that is, the ratio between the ion concentration in the membrane porewater and the bulk solution. Ξ is central to describing, for example, diffusion of ions across Mt or other types of ion exchange membranes. Figure 1 illustrates the steady state (ss) when an ion exchanger is in contact with two saline solutions of different concentrations. A cation exchanger is assumed, that is, the fixed-charged groups are negative, to make Figure 1 directly applicable to Mt. For an anion exchanger, the distribution between anions and cations in the membrane would be the reverse. Figure 1 represents a macroscopic sample. Furthermore, it is assumed that Mt on this scale is isotropic, that is, the Mt layers are equally oriented in all directions, which implies an isotropic diffusion. According to Fick’s first law, the flow of ions per unit area is proportional to the concentration gradient. In the ss, as depicted in Figure 1, the gradient  is constant through the membrane. Because of charge neutrality, cation and anion gradients must be the same in the case of salt diffusion. For cation tracers (radioactive) in a background electrolyte, the gradient may be decoupled from the anion gradient due to ion exchange, and under certain conditions, the cation tracer gradient can become very large.^26,27^ Let *D* be the diffusivity in the membrane/clay porewater and *n* the porosity; the steady-state flux is^28,29^
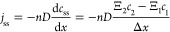
1

To calculate the concentration gradient in the membrane, the concentration jump at the interfaces must be taken into account, as given by Ξ. For ions, Ξ depends on the electrolyte concentration, hence the different values Ξ_1_ and Ξ_2_. The concentration in the membrane at the left interface is Ξ_1_*c*_1_ and correspondingly Ξ_2_*c*_2_ at the right interface (Figure 1). As illustrated in the figure, Ξ > 1 for cations (counterions), and in most cases, Ξ < 1 for anions (co-ions).

However, the ss in Figure 1 is commonly described by means of an effective diffusion coefficient, *D*_e_, that links *j*_ss_ to the concentration difference between the two solutions^26^
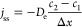
2

Note that eq 2 is not equivalent to Fick’s first law because of the imposed proportionality to an external concentration difference divided by the membrane thickness instead of an actual concentration gradient. A comparison between eqs 1 and 2 shows that in contrast to *D*, *D*_e_ is not only a property of the ion exchanger but also depends on the partition coefficients. Thus, the value of *D*_e_, evaluated from a particular test using eq 2, is not applicable to situations other than the conditions under which *D*_e_ was obtained. Using eq 2 without considering the importance of the partition coefficients can lead to incorrect prediction of the diffusive flux, both in terms of magnitude and direction, as has been demonstrated using radioactive counterion tracers and letting the nonradioactive background electrolyte to have different concentrations on the two sides of the ion exchanger.^27,29^

The ability of predicting diffusion through Mt is of practical importance for the safety assessment of a GDF because the salinity of the groundwater may change significantly during its long operating time,^30^ which may impact the partition coefficient. Furthermore, it is anticipated that Na bentonites installed in a GDF will evolve toward Ca bentonites as ion exchange occurs with calcium-rich groundwater.^2^ Thus, there is a need to broaden the focus beyond the commonly studied Na-Mt or Na-rich bentonites.^26,27,31,32^

The partition coefficient is also needed to predict how the swelling pressure of Mt responds to changes in the salinity of the external solution. An increase in salinity leads to a lowering of the water chemical potential^33^ which, in turn, leads to a lowering of the equilibrium Mt swelling pressure.^11^ However, it is now well established that the lowering of the swelling pressure is less than what would be expected by considering only the change in external water activity because some salt, determined by the partition coefficient, enters the interlayers and thereby lowers the activity of the interlayer water as well.^11,12^ As an example, assume that the swelling pressure of homoionic Mt is known for deionized water; then, to a good approximation, the change in swelling pressure Δ*p* as a result of increasing the external salt concentration to *c*_ext_ is given by^11^
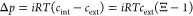
3where *c*_int_ is the internal excess salt concentration and *i*, *R*, and *T* are the van’t Hoff factor, gas constant, and the absolute temperature, respectively. Because of charge neutrality, Ξ for the salt is determined by Ξ for the anion. Noteworthily, although outside the scope of this study, cation exchange involving smectites is an important process in soils, for example, for retention of K-fertilizers and other nutrients.^34^ In this context, selectivity coefficients for the cation exchange reaction can be determined from Ξ for the cations without involving the anions.^35^

Equilibrium ion concentrations in the Mt or membrane porewater have been calculated using Donnan theory^22,23,35−37^ or the Poisson–Boltzmann (PB) equation.^38−42^ Although it can be shown that Ξ calculated with the PB equation converges to the Donnan theory result when the spacing between parallel Mt layers becomes small,^43−45^ both models rely on a continuum description of water. It is precisely at small layer separations where the atomistic nature starts to matter, for example, the discrete changes in basal distance mentioned above and the shortcoming of the PB equation to predict swelling pressures.^10^ Therefore, to investigate the validity of these equations for small layer separations, one needs to employ methods beyond the continuum treatment.

Mt in equilibrium with external bulk solution has been studied at the atomistic level with grand canonical Monte Carlo (GCMC) simulations based on classical force fields for the constituent species.^13,46−48^ In such simulations, there is no explicit bulk in contact with Mt. Instead, the clay layers are fully periodic in the lateral dimensions, and the probability of insertion or deletion of water molecules and/or ions is based on the respective chemical potential in the bulk which is determined separately. An alternative to GCMC is molecular dynamics (MD) simulation on truncated clay layers in contact with an explicit bulk solution, and this scheme was first employed to study cation exchange in Na-Mt.^49^ Subsequently, this approach has been used to investigate Donnan equilibrium in Na-Mt^45,50^ and swelling/swelling pressure.^50−53^

In our previous work, we used MD simulations to study bihydrated (2WL) Na-Mt in contact with two different explicit NaCl solutions.^50^ Bihydrated Mt is relevant because in several GDF proposals involving compacted bentonite,^2−4^ the solid volume fraction implies bihydrated and trihydrated Mt at full water saturation.^45,50^ The results of the MD simulations are consistent with those predicted by Donnan theory.^50^ Importantly, this finding is also consistent with the experiment.^11,36^ A recent study, addressing the flowback of high saline water during hydraulic fracturing, employed GCMC to investigate the equilibrium partitioning of NaCl between bulk and interlayer water.^47^ A range of Mt hydration states were investigated, and their work corroborates our earlier finding that NaCl enters 2WL Mt. Furthermore, the reported increase in the interlayer NaCl concentration with the increasing bulk concentration qualitatively obeys Donnan theory. For Ca-Mt, however, there is largely a lack of systematic diffusion or other experimental work that can shed light on the partition coefficient. In addition, as mentioned above, Na bentonites can evolve into Ca bentonites; therefore, in this work, we extended the MD simulations of Na-Mt to study the corresponding 2WL Ca-Mt/CaCl_2_ system. One of the key findings is that for all external Cl^–^ concentrations in the examined range, the interlayer Cl^–^ concentrations were significantly higher for CaCl_2_ than those for NaCl. We will demonstrate that such results agree with Donnan theory.

## Theoretical Methods

### Donnan Equilibrium

As discussed in the Introduction, Donnan equilibrium will be established when Mt is brought into contact with an external saline solution. Even in the absence of excess salt, counterions must be present inside the clay phase. For water-saturated clay, we define *c*_IL_ as the average concentration of monovalent ions needed to compensate for the clay layer charge. *c*_IL_ can thus be expressed using macroscopic quantities such as the cation exchange capacity and water content.^20,35^ If microscopic quantities are employed,^50^*c*_IL_ = −σ/*eh*, where σ denotes the clay layer surface charge density, *e* the elementary charge, and 2*h* corresponds to the average interlayer thickness.

Now, consider the equilibrium between Mt and an external saline solution. If both the bulk and the internal, that is, interlayer solutions can be treated as ideal, the Donnan potential, ϕ_D_, is enough to express internal concentrations in terms of the bulk concentrations, that is, Ξ can be calculated from ϕ_D_. In the case of non-ideality, excess free energy corrections are needed, which are straightforward to introduce as long as the electrostatics in the internal solution can be described with an averaged constant potential.^35,37,50^

For Mt in equilibrium with an external bulk electrolyte solution, the interlayer concentration of ion *i* with charge *Z*_*i*_ and bulk concentration *c*_*i*_ is  where
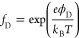
4and
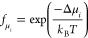
5

In eqs 4 and 5, *k*_B_ is the Boltzmann constant, and Δμ_*i*_ = μ_*i*_^bulk^ – μ_*i*_^int^ is the difference in excess chemical potential of ion *i* between the bulk and the interlayer solutions. The formalism presented here encompasses classical Donnan theory with activity corrections included^23,24,35,37^ in which case  is identified as the ratio between activity coefficients in the bulk and the interlayer solutions.^54^

Charge neutrality in the clay phase implies that the net concentration of charge must equal *c*_IL_. Using eqs 4 and 5, this charge balance can be expressed as^50^
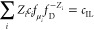
6

For a 1:1 salt, such as NaCl, with bulk concentration *c*_B_, eq 6 gives
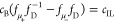
7where subscripts + and – refer to the monovalent cation and anion, respectively. When the amount of excess salt entering the interlayer is small, the Donnan factor can be evaluated to the leading order from the counterion concentration as .^35,50^ As was noted in the Introduction, the partition coefficient for excess salt can be calculated from the interlayer anion concentration . With the approximate Donnan factor, one obtains the 1:1 salt partition coefficient
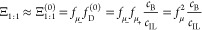
8

For the last step in eq 8, we use  which is equivalent to defining the mean value for the excess free energy difference Δμ_+_ + Δμ_–_ = 2Δμ, analogous to the mean salt method for calculating activities.^55^

For 2:1 salts, for example, CaCl_2_, eq 6 becomes
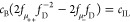
9

Equation 9 is a cubic equation in *f*_D_ and can be simplified to a quadratic equation when . Thus, to leading order,  which gives the partition coefficient for 2:1 salts as
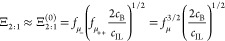
10

As in eq 8, we use Δμ for the salt obtained from the mean value: Δμ_++_ + 2Δμ_–_ = 3Δμ, from which  in eq 10 follows.

Equations 8 and 10 are valid when the interlayer anion concentration is much lower than *c*_IL_. To fulfill this condition, it is necessary that *c*_B_ < *c*_IL_ unless Δμ is very large. For 1:1 salt, the partition coefficient increases linearly with *c*_B_, while for a 2:1 salt, it increases as the square root of *c*_B_. From this difference, it follows, other factors being equal except for the cation, that the interlayer anion concentration is significantly higher for a 2:1 salt than that for a 1:1 salt at low *c*_B_.

### Molecular Model

X-ray diffraction shows that all water volume in compacted Mt is accounted for by the interlayer.^56^ Therefore, our simulations focus on the equilibrium between interlayer and external bulk solution. The molecular model in this study is identical to that in our previous work on NaCl permeation in bihydrated Na-Mt:^50^ Mt clay layers are assumed to be rigid and fixed with a basal distance of 15.4 Å. A structural charge of −0.75*e* per unit cell is placed in the octahedral sheet. Formally, this would give a structural formula of the unit cell as [Ca_0.375_/Na_0.75_]Si_8_Al_3.25_Mg_0.75_O_20_(OH)_4_ for Ca-/Na-Mt. However, instead of explicit Mg for Al substitutions, the octahedral charge is distributed evenly over all Al atoms by reducing the Al partial charge by 0.1875*e*.^45,50^ The atomic positions are assigned according to the model by Skipper et al.^57^ in which the lateral dimensions of the clay layer unit cell are 5.28 Å × 9.14 Å. Partial charges and Lennard-Jones (LJ) parameters for the Mt layer atoms and ions (Na^+^,Ca^2+^,Cl^–^) are taken from the CLAYFF force field.^58−60^ The Mt layers are terminated with −OH and −OH_2_ groups adjacent to the explicit bulk water solution. The LJ parameters for these groups are the same as those for the corresponding elements in CLAYFF, while the partial charges are adjusted based on Mulliken populations from density functional theory calculations.^50^ The partial charges for the edge atoms are presented in the Supporting Information (Table S1). The edge structure is also presented in the Supporting Information (Figure S1). Note that the present parameters for the edge atoms have not been tested for flexible Mt layers. For problems where layer flexibility may be crucial, different parameters for the edge atoms have been proposed.^53^ Water is represented with the SPC/E model^61^ and kept rigid using the SHAKE algorithm.^62^ The LJ parameters between unlike atoms are calculated according to the Lorentz–Berthelot mixing rules, that is, the arithmetic mean for the distance parameter and the geometric mean for the energy parameter.^63^ The present model for bihydrated Ca-/Na-Mt gives *c*_IL_ = 4.23 M.^50^

**Figure 2:**
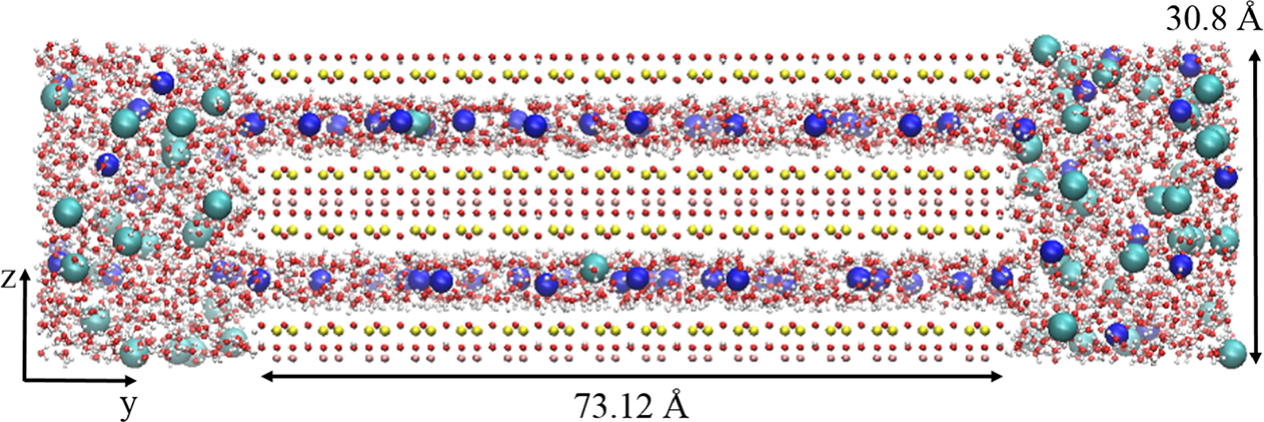
Snapshot, generated using VMD,^64^ of the simulation cell from the enhanced sampling MD simulation of bihydrated Ca-Mt in contact with 0.84 M CaCl_2_ bulk solution: Ca^2+^ (blue) and Cl^–^ (cyan).

We used the large system from our previous study with two clay layers, each consisting of 64 unit cells in an 8 × 8 arrangement (42.24 Å × 73.12 Å). The dimensions of the simulation cell, including the bulk solution, are 42.24 Å × 118.12 Å × 30.8 Å. The simulation cell and details of the interlayer solutions are shown in Figure 2. The number of water molecules, cations, and chloride ions in each of the studied systems is given in the Supporting Information (Table S2).

### Enhanced Sampling MD

Our main objective is to evaluate the equilibrium salt concentration in the interlayer as a function of the external bulk concentration. In our previous work,^50^ we compared unbiased MD trajectory sampling with results from the adaptive biasing force (ABF) method^65,66^ and found that the latter was much more efficient for calculating the partition coefficient, especially at lower external concentrations. To obtain converged trajectory sampling results for the large system, 200 ns were needed when *c*_B_ = 1.67 M NaCl, while at the lower *c*_B_ = 0.55 M, the simulation must be 10 times longer.^50^ For this reason, the ABF method is used in this work to compute the potential of mean force (PMF) from which the partition coefficient can be evaluated. For the present system, the *y*-coordinate (Figure 2) of chosen Cl^–^ is the reaction coordinate. The PMF was scanned from the middle of one interlayer (*y* ≡ 0) to one end of the simulation cell. Stratification^65^ by dividing the reaction coordinate into segments was used to ensure abundant and even sampling. A harmonic bias with a force constant of 20 kcal/mol/Å^2^ was used to confine chosen Cl^–^ within the given segment. The bin width for accumulation of the instantaneous force was 0.05 Å, and a threshold of at least 30,000 samples was set before applying any biasing force. The simulation was run for 5 ns per angstrom using a time step of 1 fs. From the PMF curves, the free energy difference Δ*A*_PMF_^Cl^ at each bulk concentration is evaluated using the difference between the average interlayer and the average bulk values. In the calculation of Δ*A*_PMF_^Cl^, the interlayer and bulk regions are chosen to be distant from the interface so that any influence from the space-charge region between interlayer and bulk solution is avoided.^50^ For the present calculations, bulk corresponds to *y* ∈ [45, 55] Å and interlayer to *y* ∈ [0, 30] Å. The average interlayer chloride concentration is given by . Hence, .

All simulations were done in the canonical ensemble using NAMD.^67,68^ The electrostatic energy was calculated using the particle-mesh Ewald method^69^ with a non-bonded cutoff distance of 10 Å, and temperature was regulated using Langevin dynamics. As in our earlier work,^45,50^ the temperature was set to 350 K, reflecting a realistic temperature in a bentonite buffer during the first 100 years of operation of a high-level radioactive waste repository.

## Results and Discussion

**Figure 3:**
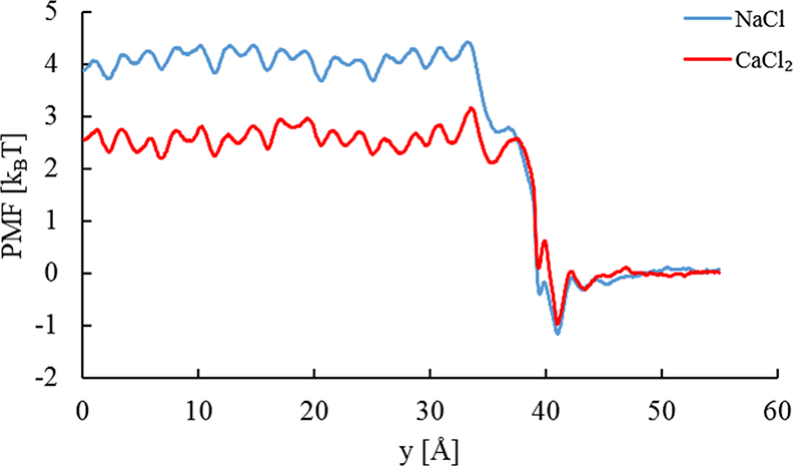
Chloride PMF profiles: comparison between NaCl and CaCl_2_ at a similar external Cl^–^ concentration, 1 M NaCl and 0.52 M CaCl_2_, respectively.

Previously, we investigated the Donnan equilibrium of 2WL Na-Mt in two bulk NaCl concentrations, 0.55 and 1.67 M.^50^ The calculation of the Na-Mt/0.55 M NaCl system is redone herein to match the stricter criteria for the ABF sampling in this work. The earlier result at 1.67 M NaCl was obtained using trajectory sampling and is deemed well-converged.^50^ To enhance the reliability of the regression of the partition coefficient to the bulk concentration, eq 8, an additional ABF calculation was made at an intermediate concentration of 1 M. Its PMF is shown in Figure 3. For comparison to the system with the divalent cation, the PMF for Ca-Mt/CaCl_2_ at *c*_B_ = 0.52 M, that is, approximately the same , is also exhibited. Each PMF curve is aligned so that the bulk average (*y* ∈ [45, 55] Å) is 0. The free energy for chloride to enter the Mt interlayer is clearly higher when Mt is in equilibrium with NaCl(aq) than with CaCl_2_(aq), in qualitative agreement with eqs 8 and 10. The average interlayer PMF is 1.5*k*_B_*T* lower for CaCl_2_, giving 4.5 times higher  compared to that of the Na-Mt/NaCl system. Noteworthily, the undulation pattern in the interlayer PMF is the same for both systems shown in Figure 3. As shown in the Supporting Information, the free energy undulation reflects the interaction among water, ions, and clay siloxane oxygens; thus, the local free energy environment for chloride is approximately the same regardless of the cation being Na^+^ or Ca^2+^ and regardless of the free energy difference Δ*A*_PMF_^Cl^.

**Figure 4:**
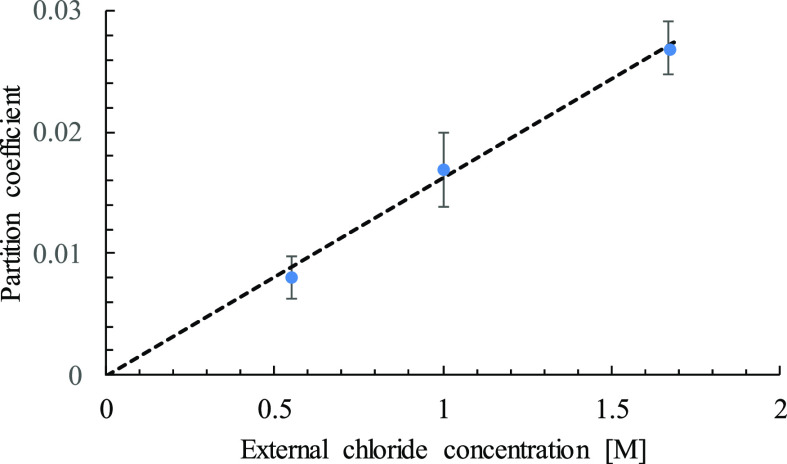
Partition coefficient of Cl^–^ in Na-Mt as a function of . The result at 1.67 M (trajectory sampling) is taken from ref (50). The partition coefficient at 0.55 and 1 M is obtained from ABF calculation of the PMF. Linear regression of the data is given by the dashed line.

Figure 4 shows the partition coefficient of Cl^–^ in Na-Mt at the three investigated NaCl concentrations. The results at *c*_B_ = 0.55 and 1 M are obtained from ABF calculations as described above, while the result at the highest concentration, 1.67 M, is calculated from trajectory sampling.^50^ The regression slope is *f*_μ_^2^/*c*_IL_ (eq 8) from which Δμ, the excess free energy, can be evaluated using eq 5. With *c*_IL_ = 4.23 M, Δμ = 1.35 *k*_B_*T* is obtained for Na-Mt/NaCl. The results are consistent with our earlier calculations and show that NaCl enters bihydrated Mt also at *c*_B_ = 0.55 M, a result that was questioned in a subsequent conventional MD study.^70^ However, the conclusion in that study that chloride is totally excluded from bihydrated Mt is based on an artifact arising from a much too short simulation time of 53 ns, that is, less than 3% of the required time for a converged result^50^ as discussed above.

**Figure 5:**
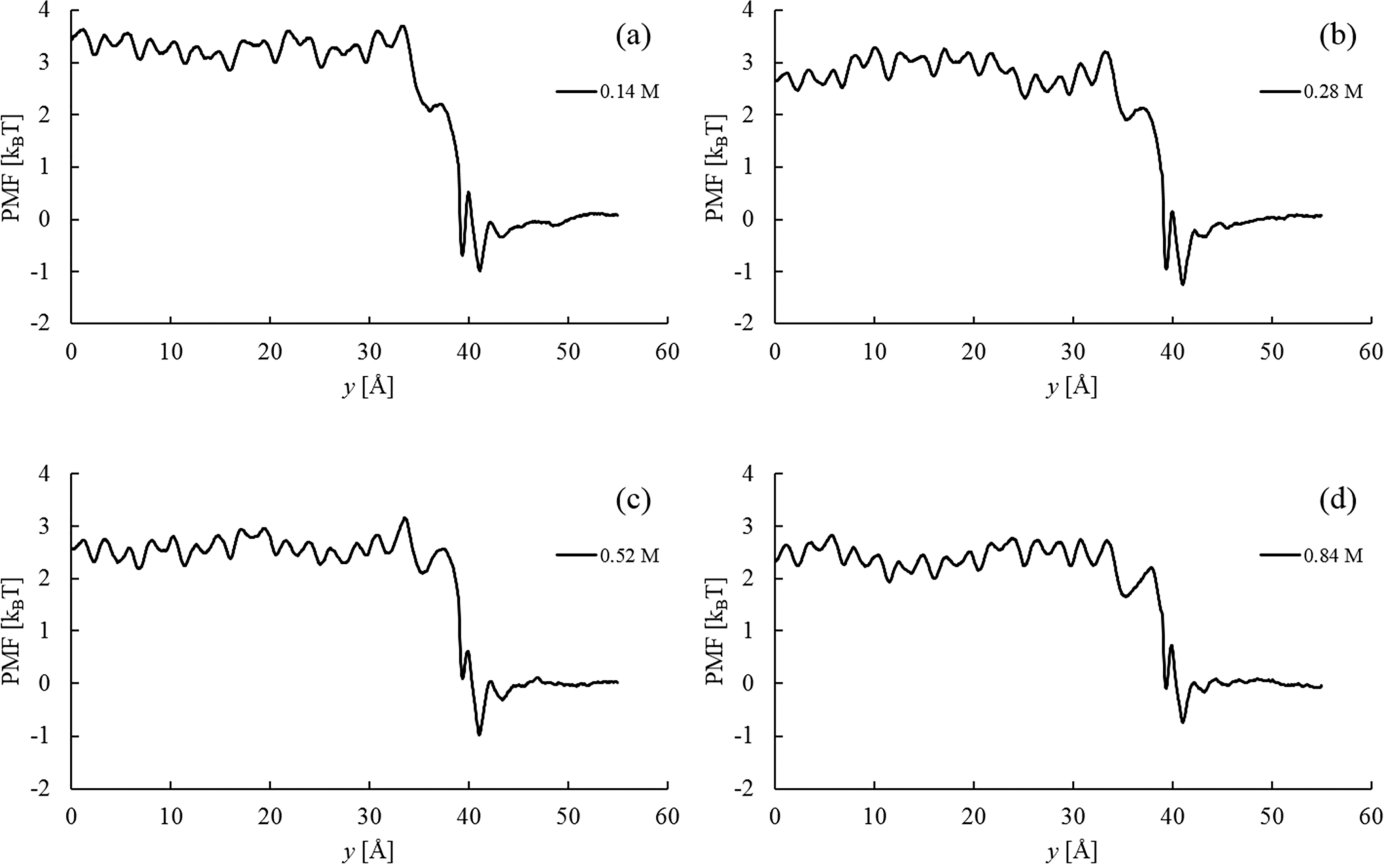
PMF profiles for Cl^–^ in Ca-Mt in equilibrium with various external CaCl_2_ bulk concentrations: (a) 0.14, (b) 0.28, (c) 0.52, and (d) 0.84 M.

Chloride PMF profiles at the studied bulk CaCl_2_ concentrations are compiled in Figure 5. Upon further observation regarding NaCl and CaCl_2_ in Figure 3, the undulation pattern in the interlayer PMFs is the same irrespective of the bulk concentration. The free energy difference, Δ*A*_PMF_^Cl^, decreases with the increasing bulk concentration and is tabulated in Table 1. The statistical error in Δ*A*_PMF_^Cl^ (Table 1) was calculated from the total variance of the PMF, that is, the sum of variances in the bulk, interlayer, and space-charge region.^71,72^

**Table 1:** Average Free Energy Difference between the Interlayer and Bulk for Cl^–^ at Different Bulk [CaCl_2_], Obtained from Figure 5^a^

[CaCl_2_] [M]	Δ*A*_PMF_^Cl^ [*k*_B_*T*]	Ξ_2:1_
0.14	3.27 ± 0.20	0.038 ± 20%
0.28	2.84 ± 0.25	0.058 ± 25%
0.52	2.57 ± 0.20	0.077 ± 20%
0.84	2.43 ± 0.25	0.088 ± 25%

a The partition coefficient is calculated using .

**Figure 6:**
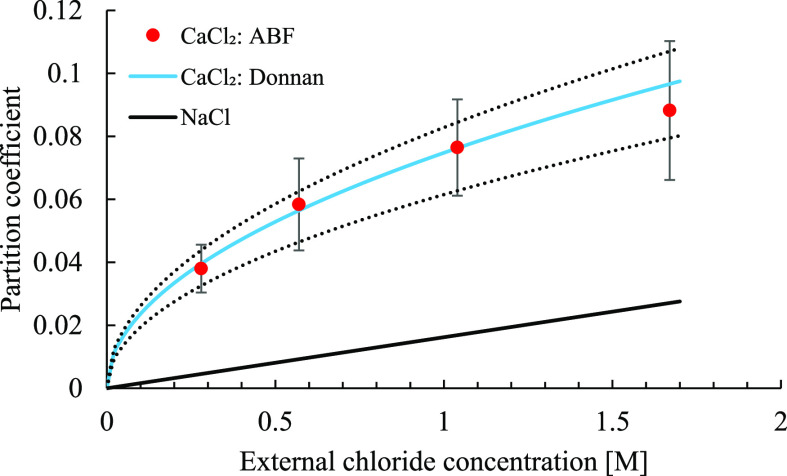
Chloride partition coefficient as a function of , demonstrating the qualitative and quantitative difference of the Donnan equilibrium for NaCl and CaCl_2_. The solid blue line is the Donnan theory result for CaCl_2_ fitted to the ABF calculations using eq 10. Dotted lines represent variations in Δμ that fit eq 10 within simulation error bars. The solid black line is the regression of the partition coefficients Na-Mt/NaCl as shown in Figure 4.

Figure 6 shows the relationship between the partition coefficient and . Results for both CaCl_2_ and NaCl are plotted for comparison. For all investigated concentrations, the partition coefficient is substantially higher for Ca-Mt/CaCl_2_ than that for Na-Mt/NaCl, and the ratio between the two increases with decreasing . In addition, the Ξ_2:1_ data do not vary linearly with . A linear fit to Ξ_2:1_ would require a constant term for the intercept, which would conflict with Ξ → 0 as *c*_B_ → 0. Instead, the data can be well fitted with the corresponding theoretical result for a 2:1 salt given in eq 10, yielding Δμ = 1.25*k*_B_*T*. The highest concentration was excluded from the fit because of the larger absolute and relative statistical uncertainty. As in the case of Na-Mt, the agreement with eq 10 demonstrates that the Donnan potential describes the electrostatics well also for 2WL Ca-Mt in equilibrium with CaCl_2_ solutions. The dotted curves in Figure 6 represent the variations in Δμ, 1.18 and 1.38*k*_B_*T*, which fit the theoretical expression, eq 10, within the simulation error bars. To confirm the validity of eq 10, exact third-order eq 9 was solved with Δμ = 1.25*k*_B_*T*: at *c*_B_ = 1.7 M, eq 10 overestimates Ξ_2:1_ by less than two percent, and for *c*_B_ ≤ 1 M, the exact solution is within the thickness of the blue line representing eq 10 in Figure 6.

The difference in excess free energy Δμ is very similar for Ca-Mt and Na-Mt. Previously, we demonstrated that Δμ was largely accounted for by the hydration free energy difference between the bulk and interlayer for NaCl.^50^ The slightly lower Δμ for CaCl_2_ in Ca-Mt might reflect the smaller difference between the first hydration shell of Cl^–^ in the bulk and interlayer in the case of Ca-Mt compared to that in Na-Mt. Coordination numbers of the first hydration shell are further discussed in the Supporting Information. Still, it should be noted that Δμ is likely to be overestimated when calculated with a non-polarizable force field.^50,73^ However, the exact value of Δμ is not critical for the discussion here. Rather, these MD simulations validate the principles of classical Donnan theory also for nanometer-sized pores: with the division of the free energy difference into an excess contribution and an electrostatic Donnan potential. It is the contribution from ϕ_D_ that determines the different behaviors of Ξ_1:1_ and Ξ_2:1_ perfectly captured in the MD simulations shown in Figure 6. For Ca^2+^ or other divalent counterions, ϕ_D_ needs not be as negative as that in the monovalent case to ensure that the layer charge is compensated, which in comparison leads to less repulsion for anions. Furthermore, because of the divalent charge, the interlayer cation concentration is proportional to *f*_D_^–2^, cf. eq 6, which is the origin of the square root increase in Ξ_2:1_ with *c*_B_.

The results in Figure 6 are obtained for Na- and Ca-Mt with the same model Mt interlayer, which means the same porosity and pore size; therefore, the difference in the interlayer Cl^–^/anion concentration is not related to differences in porosity but can be fully explained within the framework of Donnan theory. Experimentally, these predictions could be tested by performing diffusion tests on 2WL Ca- and Na-Mt, and analysis of the results using eq 1 has already been done for chloride salt diffusion through 3WL Mt.^74^ Alternatively, the same types of clays could be equilibrated with different concentrations of NaCl or CaCl_2_ and the chloride content in the clay phase analyzed.

## Conclusions

Donnan equilibrium between bihydrated Mt and external salt solution was examined using enhanced sampling MD simulations. Two types of systems were studied, Na-Mt/NaCl and Ca-Mt/CaCl_2_, for a range of external salt concentrations. The partition coefficient, that is, the ratio between the interlayer and external salt (Cl^–^) concentrations, was determined from the potential of mean force for Cl^–^ to enter the interlayer. For all examined external concentrations, the partition coefficients were larger for Ca-Mt/CaCl_2_ than those for the corresponding Na-Mt/NaCl system. In the case of Na-Mt, the partition coefficient increased linearly with the increasing NaCl concentration in the external solution. For Ca-Mt, the increase was proportional to the square root of the CaCl_2_ concentration. Both results are consistent with classical Donnan theory, thus demonstrating its validity for narrow nanometer-sized pores. Although one would arrive at the same conclusion starting from the PB equation,^43^ the present results are more robust since the atomistic nature of the interlayer is accounted for. Furthermore, we observed that the undulations in the interlayer PMF for Cl^–^ were independent of the external salt concentration and type of cation. To understand the reason behind the density profiles of Cl^–^ and Ca^2+^, water oxygen and hydrogen were analyzed, which showed that the undulations of all species were correlated with the hydrogen bonding network to the siloxane oxygens of the Mt layer (Supporting Information). Based on this, we conclude that the interlayer environment is largely independent of the external salt concentration which suggests that the diffusion coefficients in the clay phase would be independent of the concentration as well. This implies that the dependence of the external salt concentration on the ss diffusion through compacted Mt is determined by the partition coefficient, obtainable from Donnan theory as demonstrated in this work. Consequently, this provides significant simplification and clearer physical understanding when analyzing diffusion experiments.

## Supporting Information Available

Partial charges and positions of the edge atoms; number of water molecules, cations, and chloride ions in the studied systems; and interlayer structure (PDF)
