# Supplementary material for: Salt Effect on Donnan Equilibrium in Montmorillonite Demonstrated with Molecular Dynamics Simulations
Source: J Phys Chem B. 2022 Oct 24;126(43):8873–81. doi: 10.1021/acs.jpcb.2c04016 (PMC9639135; doi:10.1021/acs.jpcb.2c04016)
Supplement: Supplementary file 1 — jp2c04016_si_001.pdf [file jp2c04016_si_001.pdf]

Salt Effect on Donnan Equilibrium in Montmorillonite Demonstrated with Molecular Dynamics Simulations

Ya-Wen Hsiao† and Magnus Hedström*‡*

†Scientific Computing Department, STFC Daresbury Laboratory, Daresbury WA4 4AD, U.K.

‡Clay Technology, Ideon Science Park, SE-223 70, Lund, Sweden

*Corresponding authors: ya-wen.hsiao@stfc.ac.uk, mh@claytech.se

Supporting Information

Partial Charges and Positions of the Edge Atoms

Table S1 Partial charges for the edge atoms in montmorillonite model used in the calculations.

Atom	Type	Partial charge (e)
Si	$-\mathbf{Si}-\mathrm{OH}$	2.0400
O	$-\mathrm{Si}-\mathbf{O}\mathrm{H}$	-0.8500
H	$-\mathrm{Si}-\mathrm{O}\mathbf{H}$	0.3850
Al	$-\mathbf{Al}\begin{smallmatrix}\nearrow \mathrm{OH}\\ \searrow \mathrm{OH_2}\end{smallmatrix}$	1.3075
O	$-\mathrm{Al}-\mathbf{O}\mathrm{H}$	-0.9500
H	$-\mathrm{Al}-\mathrm{O}\mathbf{H}$	0.4250
O	$-\mathrm{Al}-\mathbf{O}\mathrm{H_2}$	-0.6900
H	$-\mathrm{Al}-\mathrm{O}\mathbf{H_2}$	0.3850

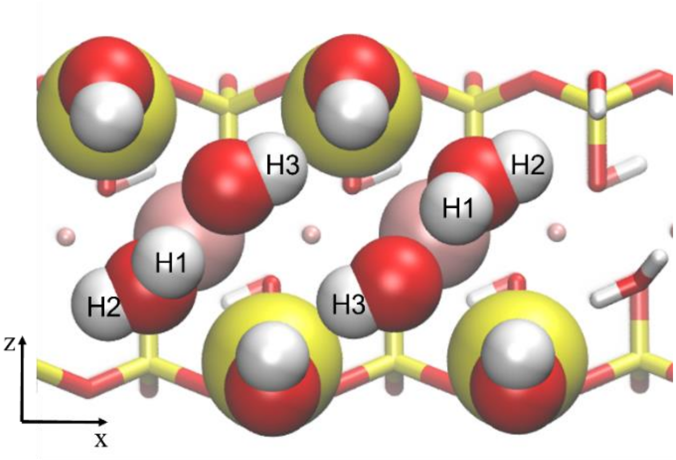

Figure S1 Detail of the edge structure showing the terminating OH and OH_2 groups. Color scheme: Si (yellow), Al (pale pink), O (red), and H (white).

The edge structure is shown in Figure S1. The coordinates of Si, Al, and O at the edge are the same as in the periodic crystal structure.[1] Further, the hydrogens labelled H2 and H3 in Figure S1 are also at the same positions as the corresponding hydrogens in the crystal. Regarding the Si-OH group: The O-H bond length is 0.97 Å and the Si-O-H bond angle is 117.28°. Regarding the Al-OH and Al-OH_2 groups: All O-H bond lengths are 0.96 Å. The Al-O-H1 bond angle is 95.49° and the Al-O-H2 bond angle is 126.64°. The dihedral angle, H1-O-Al-H2 is 144.9°.

Table S2 Number of water molecules, cations, and chloride ions in the studied systems.

System	#H_20	#Na^+	#Ca^{2+}	#Cl^-
0.55 M NaCl	3151	115	-	19
1 M NaCl	3112	131	-	35
1.67 M NaCl	3112	154	-	58
0.14 M $CaCl_2$	3164	-	53	10
0.28 M $CaCl_2$	3151	-	58	20
0.52 M $CaCl_2$	3112	-	66	36
0.84 M $CaCl_2$	3112	-	77	58

Interlayer Structure

In this section, we would like to discuss the undulations seen in the chloride PMF profiles. As noted in the main text, the chloride PMF profiles along the y-direction, show the same undulation pattern in the interlayer, irrespective of the type of counterion and free energy difference $\Delta A_{\mathrm{PMF}}^{\mathrm{Cl}}$. We observed that the undulations are primarily correlated with those of the interlayer water density profile, which are linked to the positions of siloxane oxygens through hydrogen bonds. To illustrate this, we did MD trajectory sampling of Ca-Mt/0.84 M $CaCl_2$ and evaluated $-\ln(\rho_y/\rho^*)$ for Cl^-, Ca^{2+}, water oxygens (O_w) and hydrogens (H_w) from the respective density profiles ρ_y. The results for the interlayer region are shown in Figure S2. For Ca^{2+} and O_w, ρ^* was taken as the bulk density, while for Cl^-, for clearer comparison with the other curves, ρ^* is scaled so that $-\ln(\rho_y/\rho^*)$ is shifted down to oscillate around +1. The H_w curve has also been slightly shifted to avoid overlap with the O_w profile. Since $-\ln(\rho_y/\rho^*) = \mathrm{PMF}/k_BT$ we refer to $-\ln(\rho_y/\rho^*)$ as the PMF curve from now on. To relate these PMF curves to the Mt structure, the projection of the siloxane oxygen density onto the

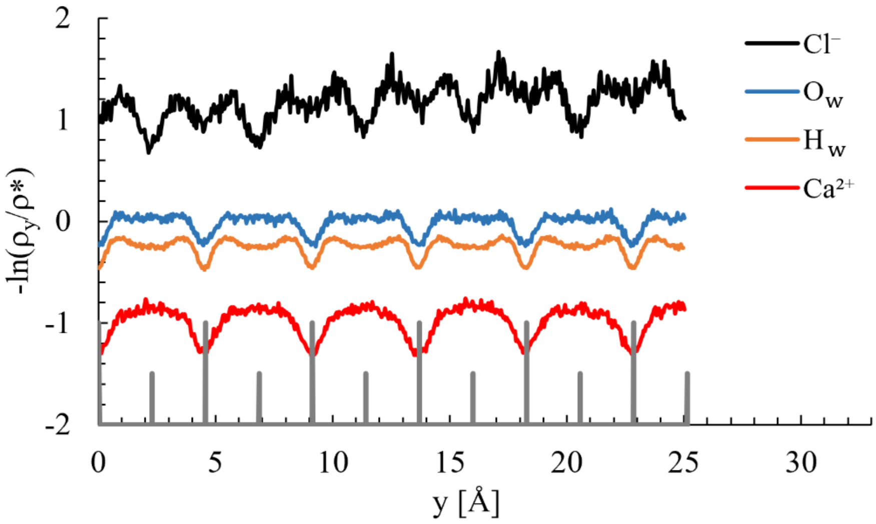

Figure S2 Undulation pattern of the PMF along y in the interlayer for Cl^{-}, water O_w and H_w, and Ca^{2+}, respectively, as evaluated from trajectory sampling. The grey vertical bars represent the projection of the siloxane oxygen density onto the y-axis. The repeat distance is 4.57 Å and the siloxane oxygens forms two groups where one peak is twice as high as the other.

y-axis is indicated as vertical grey bars at the bottom of Figure S2. In the Mt structure, the siloxane oxygens form two groups where one is twice as many as the other with respect to the y-axis, resulting in two different peak heights. They reflect the hexagonal structure of the surface as indicated in Figure S3: the smaller group is at the center of the hexagon and the other is at the edge. The repeat distance is 4.57 Å which is half of the clay layer unit cell.

The PMF curve for H_w shows two types of minima: one sharper and deeper and coincident with the larger siloxane oxygen peaks and the other shallow and coincident with the smaller siloxane oxygen peaks. The PMF for water oscillates around zero as shown by the unshifted O_w PMF, so on average the water density in the bihydrated interlayer is approximately the same as in bulk.[2] The PMF of O_w shows only one type of minimum that is synchronized with the deeper minimum in the curve for H_w. The peak positions of the PMF for Ca^{2+} look overall

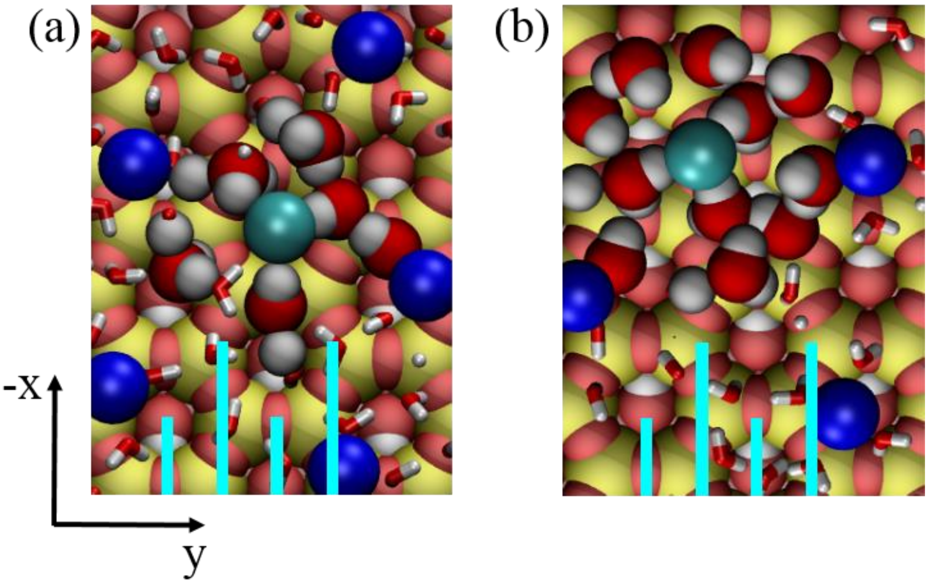

Figure S3 Top views of Cl^-—H_2O coordination for locations relating to the local minima of the Cl^- PMF. For clarity, only one layer of H_2O closest to the surface is shown. The cyan bars at the bottom of the figures indicate the siloxane oxygen density, c.f., Figure S2. Spheres represent atoms with color code pale red: siloxane oxygen; pale yellow: Si; blue: Ca^{2+}; cyan: Cl^-; red: water oxygen whose hydrogen coordinated to Cl^-. a) Cl^- above a hexagonal cavity. b) Cl^- positioned on one of the hexagonal edges.

similar to those for O_w which is expected for an ion that coordinates to eight waters.[3] Chloride coordinate to H_w and its PMF curve also displays two types of minima that coincide with the two siloxane oxygen peaks. The slightly deeper minimum corresponds to configurations where Cl^- is located above the hexagonal cavity (Figure S3 a) and the other relates to configurations where Cl^- is located on the edge of the hexagonal cavity (Figure S3 b). In conclusion, the ions are hydrated, and their hydration shells are correlated with the siloxane oxygens through hydrogen bonds, which explains the observed undulations in the calculated PMFs. Although the oscillations in ρ_y discussed above, most probably cannot be detected experimentally, especially not in a macroscopic sample, the fact that they do not

depend on c_B suggests that the diffusion coefficient D in eq 1 in the main text could be treated as independent of c_B.[4] Thus, the complexity of fitting eq 1 to experiment is reduced.[5]

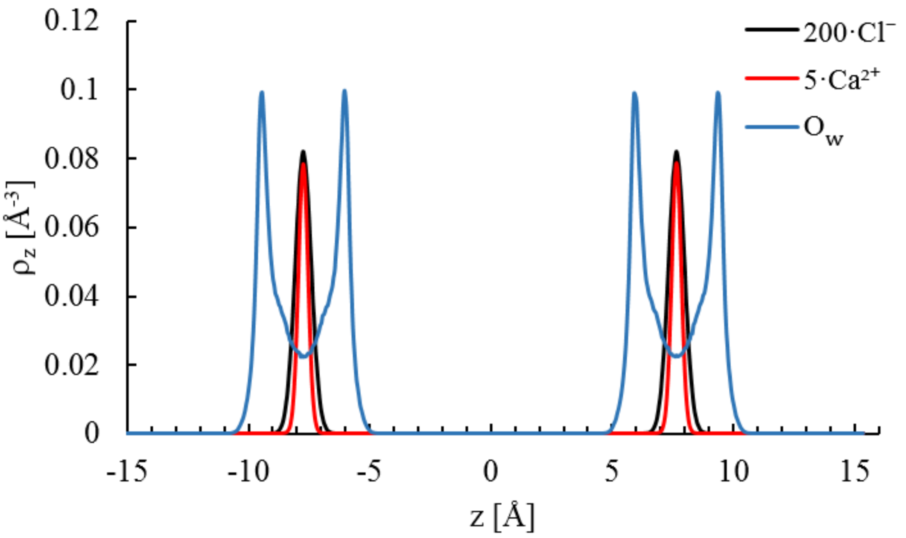

Figure S4 Densities of Cl^-, Ca^{2+}, and water oxygens in the interlayer along the z-direction obtained from trajectory sampling of a system with C_B = 0.84 M. The densities for Cl^- and Ca^{2+} have been multiplied by factors of 200 and 5, respectively.

The significance of the hydration shell around the ions is further demonstrated by ρ_z, the density profiles perpendicular to the mineral layers, shown in Figure S4. Water oxygens form a double peak reflecting the bihydrated structure, while both anions and cations are perfectly centered in the middle of the interlayer just as in the case of Na-Mt,[6,7] i.e., all species share the same pore space. Hence, there is no basis for assuming different pore spaces for anions and cations[8] or to assume that anions are excluded from the interlayers.[9]

Additionally, first-shell water coordination numbers (CN) obtained from the radial distribution functions, RDF, are very similar in bulk solution and interlayer for both cations and anions. Comparisons for cations in interlayer and bulk have been presented in a recent study.[10] Our results for Na^+ and Ca^{2+}, employing the same force field and water model, are in excellent agreement. In 2WL Ca-Mt, the Cl^--H_w RDF gives CN = 7.0 while the corresponding bulk value was 7.1. Similarly, the Ca^{2+}-O_w RDF gives CN = 7.9 in both

environments. In addition, the respective RDF peaks at the same distance in bulk and interlayer, giving $r_{Cl^- - H_w} = 2.25$ Å and $r_{Ca^{2+} - O_w} = 2.45$ Å. For comparison, the Na-Mt/NaCl system from our previous study[6] gave $r_{Cl^- - H} = 2.25$ Å and $r_{Na^+ - O_w} = 2.35$ Å. Na^+-O_w CNs were 5.9 in both interlayer and bulk, whereas for Cl^--H_w the CN showed larger difference, 6.3 and 7.1 in interlayer and bulk, respectively. This reduction in CN for interlayer Cl^- in Na-Mt is the only notable difference at the atomistic level between the Ca- and Na-Mt systems. A possible explanation is that more water molecules are required to hydrate the twice as many cations in Na-Mt compared to Ca-Mt. This leaves fewer water molecules for hydrating the excess salt ions, and seemingly a reduced CN for Cl^- is favorable.

References

(1) Skipper, N. T.; Chang, F.-R. C.; Sposito, G. Monte Carlo Simulation of Interlayer Molecular Structure in Swelling Clay Minerals; 1, Methodology. *Clays Clay Miner.* **1995**, *43*, 285–293.

(2) Hsiao, Y.-W.; Hedström, M. Swelling Pressure in Systems with Na-Montmorillonite and Neutral Surfaces: A Molecular Dynamics Study. *J. Phys. Chem. C* **2017**, *121* (47), 26414–26423.

(3) Jalilehvand, F.; Spångberg, D.; Lindqvist-Reis, P.; Hermansson, K.; Persson, I.; Sandström, M. Hydration of the Calcium Ion. An EXAFS, Large-Angle X-Ray Scattering, and Molecular Dynamics Simulation Study. *J. Am. Chem. Soc.* **2001**, *123* (3), 431–441.

(4) Birgersson, M.; Hedström, M.; Karnland, O.; Sjöland, A. 12 - Bentonite Buffer: Macroscopic Performance from Nanoscale Properties. In *Geological Repository Systems for Safe Disposal of Spent Nuclear Fuels and Radioactive Waste (Second Edition)*; Woodhead Publishing Series in Energy; Woodhead Publishing, 2017; pp 319–364.

(5) Birgersson, M. A General Framework for Ion Equilibrium Calculations in Compacted Bentonite. *Geochim. Cosmochim. Acta* **2017**, *200*, 186–200.

(6) Hsiao, Y.-W.; Hedström, M. Molecular Dynamics Simulations of NaCl Permeation in Bihydrated Montmorillonite Interlayer Nanopores. *J. Phys. Chem. C* **2015**, *119*, 17352–17361.

(7) Moučka, F.; Svoboda, M.; Lísal, M. Modelling Aqueous Solubility of Sodium Chloride in Clays at Thermodynamic Conditions of Hydraulic Fracturing by Molecular Simulations. *Phys. Chem. Chem. Phys.* **2017**, *19* (25), 16586–16599.

(8) Altmann, S.; Tournassat, C.; Goutelard, F.; Parneix, J.-C.; Gimmi, T.; Maes, N. Diffusion-Driven Transport in Clayrock Formations. *Appl. Geochem.* **2012**, *27* (2), 463–478.

(9) Bradbury, M. H.; Baeyens, B. Porewater Chemistry in Compacted Re-Saturated MX-80 Bentonite. *J. Contam. Hydrol.* **2003**, *61* (1–4), 329–338.

(10) Li, X.; Liu, N.; Tang, L.; Zhang, J. Specific Elevated Adsorption and Stability of Cations in the Interlayer Compared with at the External Surface of Clay Minerals. *Appl. Clay Sci.* **2020**, *198*, 105814.
